# Protective Effects of Donkey Milk on Ethanol‐Induced Gastric Ulcer in Rat

**DOI:** 10.1002/vms3.70156

**Published:** 2024-12-12

**Authors:** Masoud Sami, Shahrzad Azizi, Reza Kheirandish, Hadi Ebrahimnejad, Shiva Alizadeh

**Affiliations:** ^1^ Department of Food Science and Technology School of Nutrition and Food Science Nutrition and Food Security Research Center Isfahan University of Medical Sciences Isfahan Iran; ^2^ Department of Pathobiology Faculty of Veterinary Medicine Shahid Bahonar University of Kerman Kerman Iran; ^3^ Department of Food Hygiene and Public Health Faculty of Veterinary Medicine Shahid Bahonar University of Kerman Kerman Iran

**Keywords:** ethanol, gastric ulcer, malondialdehyde, rat

## Abstract

Gastric ulcer (GU) is the most common health concern that occurs due to an imbalance between gastric protective mucosal and aggressive factors. Ethanol‐induced GU in animal models resembles the pathophysiology of human ulcers. Natural products with fewer side effects are highly requested to attenuate their GU effects. The present study was conducted to investigate the potential protective effects of donkey milk against ethanol‐induced GU in rats. The male Wistar were divided into four groups, including normal control (distilled water), donkey milk (1 cm^3^/animal) and ranitidine (200 mg/kg). Donkey milk and ranitidine were given to rats orally daily for 10 consecutive days before induction of ulcer by ethanol. After 24 h of fasting, GU was induced by oral administration of ethanol. After an hour, the rats were sacrificed, and gastric samples were taken for pathologic analysis, malondialdehyde (MDA) and glutathione (GSH) assessments. The results showed that the severity of ethanol‐induced gastric damage was significantly reduced by donkey's milk pretreatment and then ranitidine. Reduction of ulcer score and MDA level, and also increasing GSH in the gastric tissue in comparison with other groups supports our results. This study described the gastroprotective and antioxidative effects of donkey milk that were determined with ulcer inhibition percentage.

## Graphical abstract

We investigated the gastroprotective activity of donkey milk on ethanol‐induced gastric ulcer in rat. Results of our study indicated donkey milk significantly decreased gastric ulcer index and malondialdehyde (MDA) level in the gastric tissue. The present study suggests that one of the anti‐ulcerogenic efficacies of donkey milk may be related to its antioxidative properties. Donkey milk may exert its protective property by inhibiting lipid peroxidation marker (MDA). However, the precise mechanism for this action remains to be elucidated.

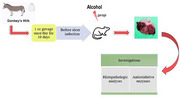

## 1 Introduction

Gastric ulcer (GU) disease is a common gastrointestinal disorder that is characterized by disruption of the mucosal integrity (Ozbayer et al. 2014). It is associated with social and economic implications and influences life quality (Hajrezaie et al. 2012). Clinical signs of the GU may appear as heartburn, nausea, stomach pain and occasionally may lead to perforation, bleeding and loss of weight if not treated (Ramakrishnan and Salinas 2007).

The aetiology of GUs is multifactorial and exactly unknown. The pathophysiology of gastric mucosal injury is mainly due to the imbalance between destructive and protective factors of the mucosal barrier (Chen et al. 2015). Several destructive factors are responsible for gastric ulcerogenesis such as acid and pepsin secretion, defect in mucosal blood supply, free oxygen radicals, alcohol, stress*, Helicobacter pylori* and prolonged usage of nonsteroidal anti‐inflammatory drugs (NSAIDs) that promote the gastric mucosal injury and contribute to ulcer formation (Saxena and Singh 2011). These harmful factors break gastric defence mechanisms such as secretion of mucus and bicarbonate, mucosal perfusion, prostaglandins, levels of antioxidants, anti‐inflammatory compounds and nitric oxide (NO) (Laine, Takeuchi, and Tarnawski, 2008).

Preventing or treating GUs is a significant challenge in the field of medical science (Okayama et al. 2009). Current treatments primarily focus on reducing acid secretion (Ritter et al. 2014) but these chemical drugs can have side effects (de Lira Mota et al. 2009).

Natural products such as milk can act as a rich source of potent therapeutics (Ononye et al. 2013). There are various functional components, including vitamins (E and C), proteins, carotenoids and flavonoids with antioxidant properties in milk. Therefore, milk with a higher antioxidant capacity causes potentially greater protection for the consumer against the oxidative stress that is a significant aspect of many acute and chronic diseases (Valko et al. 2007). In recent years, there has been a growing interest in nutritional and biofunctional properties of goats’ and donkeys’ milk (Amati et al. 2010). Donkey milk is different from the milk of cows, sheep and goats traditionally used for human feeding as it has high similarity to human milk (Guo et al. 2007). In Europe, there is a rising interest in using donkey's milk as an alternative to breast milk and for children with cow milk protein allergy (CMPA) (Mansueto et al. 2013).

To the best of the author's information, there was little information available in the literature about anti‐ulcer activities of the donkey milk. The present study was conducted to evaluate the protective effect of donkey milk against GU induced by ethanol in rat.

## 2 Materials and Methods

### 2.1 Experimental Animals and Grouping

In this study, 36 male Wister rats weighing (200–250 g) were used. The animals were housed in standard environmental conditions, including temperature (22°C ± 3°C) and 12 h light/dark cycles. They had free access to water and a standard pellet diet (Pastor Institute, Iran) ad libitum. After 1 week of compatibility, the rats were randomly divided into 4 groups (*n* = 9 for each group), including Group I: normal control (received only distilled water), Group II: ulcerated control (ethanol 96%), Group III: ranitidine as positive control group (200 mg/kg for 10 days before ulcer induction) and Group IV: donkey milk (1 mL/animal/once/day for 10 days before ulcer induction). The selected dose of milk was based on the study of Yoo et al. (2018). In addition, according to the stomach capacity of rats, gavage of higher volumes of more than 1 mL increases the chance of aspiration pneumonia associated with passive reflux of the material into the oesophagus (Turner et al. 2011).

In each group, five samples were used for pathologic study and four samples for MDA measurement in gastric tissue. The rats fasted for 24 h before induced ulcer and were supplied with sucrose 8% during the fasting period. Each animal received 1 mL of alcohol (96%) orally (Al Asmari et al. 2016).

### 2.2 Determination of Ulcer Index (UI)

Experimental rats were euthanized and then necropsied 1 h after induced ulcer. After the excision of the abdomen, the stomach was opened along the greater curvature and washed with normal saline. Ulcer areas on the glandular and non‐glandular gastric mucosa were inspected grossly. Digital photographs were taken from all wounds with a 10‐megapixel camera. Image analyser (ImageJ) was used for measuring wound size. The sum of the ulcerous areas was expressed in mm^2^ as the ulcer score in each animal. The mean ulcer score for each group was expressed as UI. The inhibition per cent against ulceration was determined using the following formula (Sabiu et al. 2015):

$$\%\mathrm{Ulcer}\mathrm{inhibition}\frac{U.I.\mathrm{in}\mathrm{control}-U.I.\mathrm{in}\mathrm{test}}{U.I.\mathrm{in}\mathrm{control}}\times 100$$

### 2.3 Pathologic Investigation

Gastric samples of the different groups were fixed in the 10% neutral buffered formalin. Then, the samples were dehydrated in graded ethanol and embedded in paraffin. Sections in 5 µm thickness were stained with haematoxylin‐eosin and investigated by an ordinary light microscope (Olympus BX51). A semiquantitative scoring system was used for histopathologic evaluations based on ulcer depth as follows (Andrews et al. 2002):

Score 0: normal
Score 1: superficial mucosal lesions
Score 2: deeper erosions extending to the submucosal layer
Score 3: deep lesion involving the tunica muscularis
Score 4: lesions extending to the tunica serosa

### 2.4 Assessment of Malondialdehyde (MDA) Formation

MDA is one of the most important products of lipid peroxidation. Thiobarbituric acid (TBA) assay kit (Kiazist, Iran) was used for determination of MDA by manufacturer instruction. An amount of 20 mg gastric tissue was promptly removed and rinsed in cold saline. To minimize the possibility of interfering haemoglobin with free radicals, any adhered blood to the mucosal layer was carefully washed. The mucosa was scraped, weighed and homogenized for estimation of lipid peroxidation. This method is based on the reactivity of MDA with the TBA to produce a chromophore complex. Results are expressed as nanomoles of MDA per gram of tissue.

### 2.5 Determination of Glutathione (GSH) Activities in Rat Stomach Tissues

Ellman reagent 5,5‐dithiobis‐(2‐nitrobenzoic acid) known as dithionitrobenzoic acid (DTNB) reacts with reduced or free sulfhydryl (–SH) groups and results in a DNTB‐sulfhydryl complex which is detectable at 405 nm (Riddles et al. 1983). Briefly, cell lysates were prepared and centrifuged, and the supernatants were used for the experiment according to the Kiazist kit instructions. The results were normalized and expressed as nanomole per milligram of protein.

### 2.6 Statistical Analysis

The mean UI, wound area and MDA level were analysed statistically by one‐way ANOVA and followed by post hoc Duncan test. Results were expressed as mean ± SEM. All statistical tests were carried out by the SPSS version 16. *p* ≤ 0.05 was considered statistically significant value.

## 3 Results

### 3.1 Macroscopic Evaluation of Gastric Lesions

Gastric sections of the normal control group displayed an intact structure of the gastric wall without any damages such as haemorrhage, ulcers and erosions.

In the ethanol group, the oral administration of alcohol resulted in severe gastric destruction mainly in the glandular part of the stomach. Extensive erosive and ulcerative lesions in different shapes and sizes were observed in the tunica mucosa. The margins of ulcers were swollen and edematous, and some of the ulcerated lesions were covered with blood clot and tissue debris. The gastric mucosa showed remarkable hyperaemia and also haemorrhage in linear and petechial forms.

**FIGURE 1:**
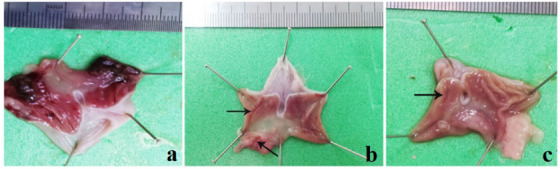
Protective effect of donkey milk on the gross appearance of ethanol‐induced gastric mucosal injuries in a rat mode. (a) Ethanol group exhibits extensive haemorrhagic ulcers in the stomach mucosa. Pretreatments with ranitidine (b) and donkey milk (c) have significantly attenuated gastric ulcers. Few linear lesions (arrows) are visible in the glandular part of stomach.

**FIGURE 2:**
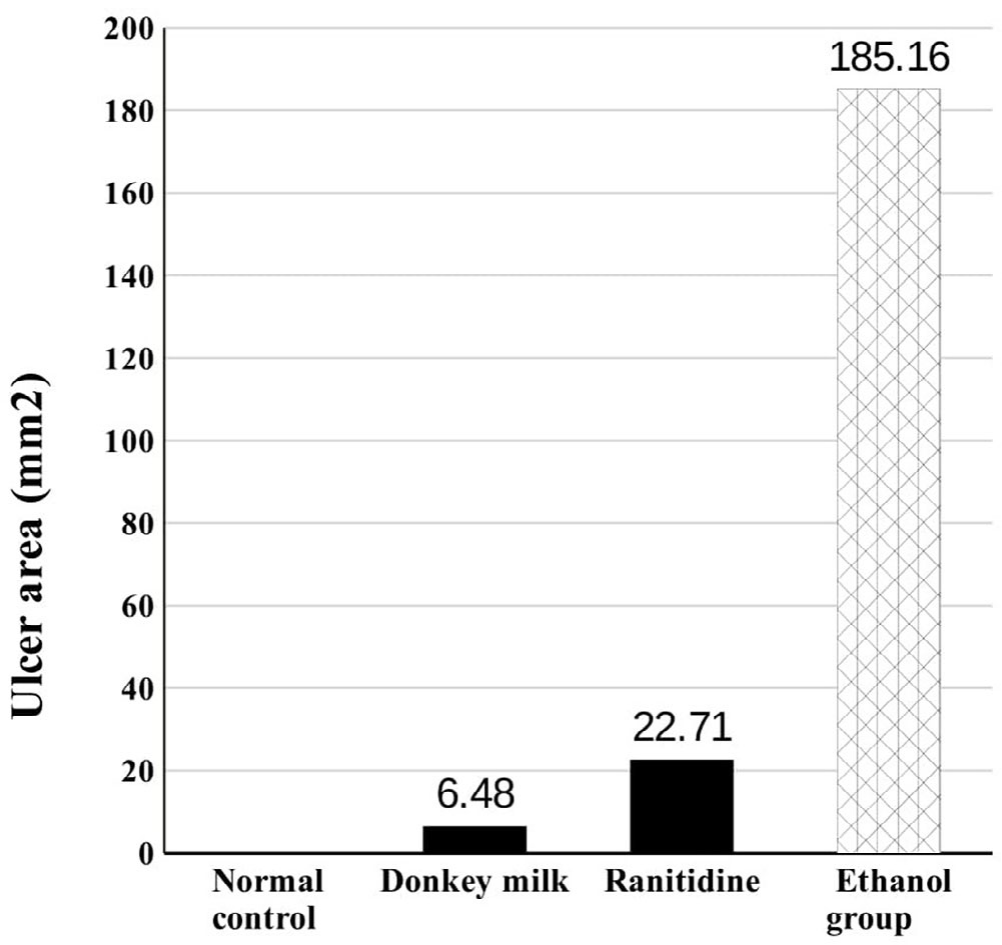
Effect of donkey milk and ranitidine on wound area in ethanol‐induced gastric ulcer in rat. Significant difference (*p* < 0.05) is present between both pretreatment groups with ethanol group.

**FIGURE 3:**
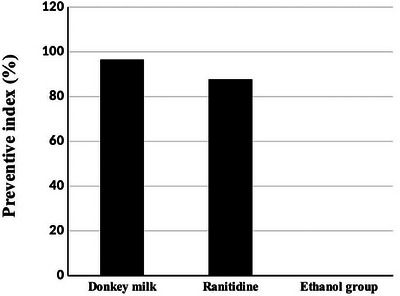
Effect of donkey milk and ranitidine on the preventive index (%) in ethanol‐induced gastric ulcer in rat (*n* = 5). Significant difference (*p* < 0.05) is present between the pretreatment groups with the ethanol group.

Pretreatment with donkey milk and ranitidine attenuated significantly alcohol‐induced lesions, including mucosal hyperaemia, haemorrhage and ulcers compared to the ethanol group (Figure 1). The ulcer score in the ethanol group was 185.16 ± 31.4. Pretreated with donkey milk and ranitidine showed a significant decrease of ulcer scores which were 6.48 ± 1.65 and 22.71 ± 5.45, respectively. Donkey milk was more effective in gastric protection with an inhibited UI (96.5%) as compared to the ranitidine (87.7%) group (Table 1) and (Figures 2 and 3).

**TABLE 1:** Anti‐ulcer effect of donkey's milk and ranitidine on the ulcer area, malondialdehyde (MDA) and glutathione (GSH) levels in ethanol‐induced gastric ulcer in rat.

Groups	Ulcer area (mm^2^)	Preventive index (%)	Malondialdehyde level (nmol/mg)	GSH level (nmol/mg)
Normal control	0.0	0.0	8.23 ± 1.05^a^	7.86 ± 0.47^a^
Donkey milk	6.48 ± 1.65^a^	96.5	28.58 ± 3.13^ab^	6.23 ± 0.78^a^
Ranitidine	22.71 ± 5.45^a^	87.7	20.16 ± 2.35^a^	4.53 ± 0.29^b^
Ethanol	185.16 ± 31.4^b^	—	40.41 ± 3.09^b^	4.81 ± 0.32^b^

*Note*: Data are expressed as mean ± SE. Different letters above the data show significant difference (*p* ≤ 0.05).

### 3.2 Malondialdehyde Measurement (MDA) in Gastric Mucosa

**FIGURE 4:**
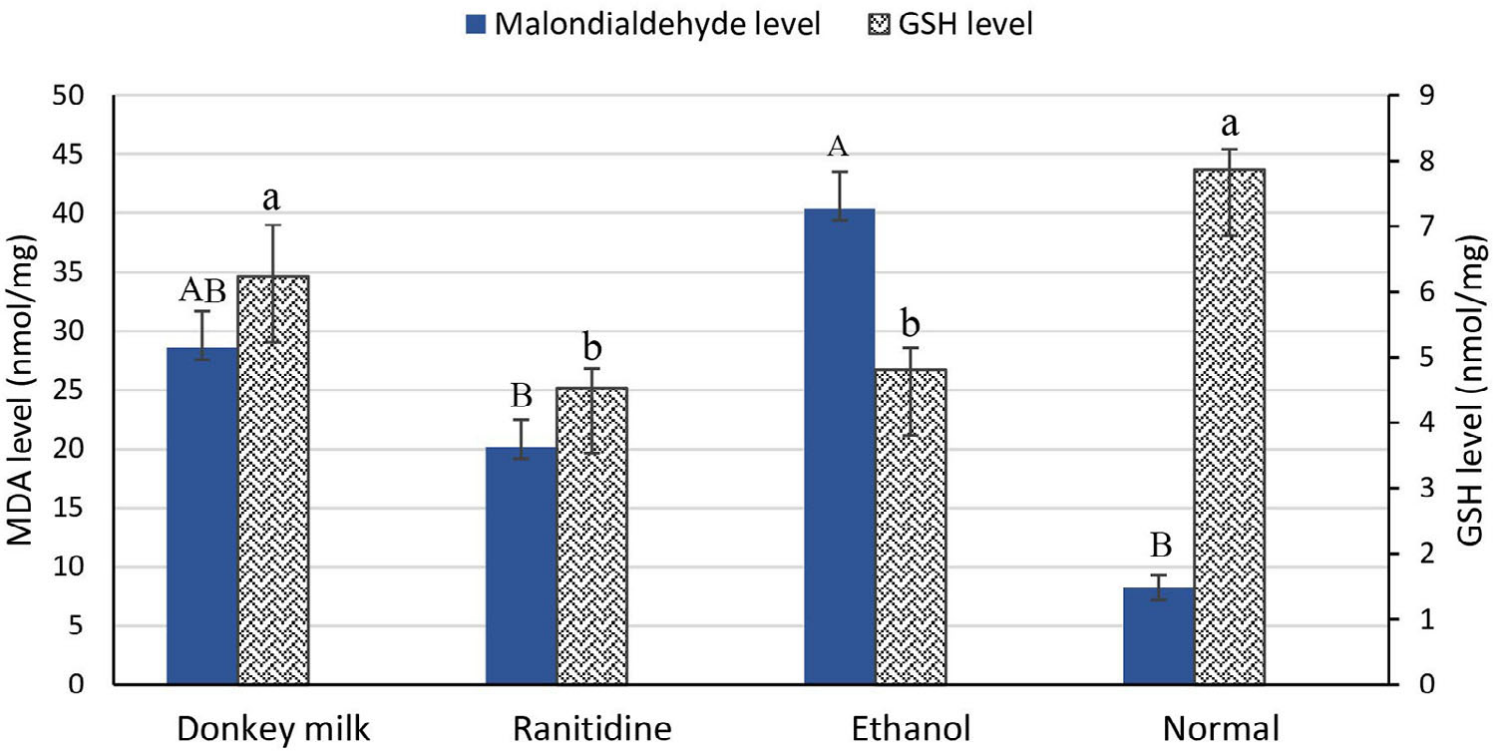
Effect of donkey milk and ranitidine on gastric tissue level of malondiaidehyde and GSH as oxidative stress parameters in the ethanol‐induced gastric ulcer in rat. Different letters indicate statistically significant difference (*p* ≤ 0.05). GSH, glutathione; MDA, malondialdehyde.

The MDA is a marker of lipid peroxidation and membrane lipid damage. In this study, ethanol administration caused a significant increase in the MDA level of the gastric mucosa (40.41 ± 3.09) compared with the ranitidine pretreatment animals (20.16 ± 2.35) (*p* ≤ 0.05) as illustrated in (Figure 4) and (Table 1).

### 3.3 GSH Activities in Rat Stomach Tissues

Antioxidant level of GSH was evaluated in order to explore effects of antioxidant defences on the ulceration progress in gastric tissues. The result showed that GSH level in the donkey's milk group was significantly higher than those in the ranitidine and ethanol groups (*p* < 0.05) (Figure 4 and Table 1).

### 3.4 Microscopic Evaluation of the Gastric Lesions

Normal control rats showed intact gastric mucosa with no signs of haemorrhages or congestion. Moreover, there is no destruction and exfoliation in the mucosal epithelium.

**FIGURE 5:**
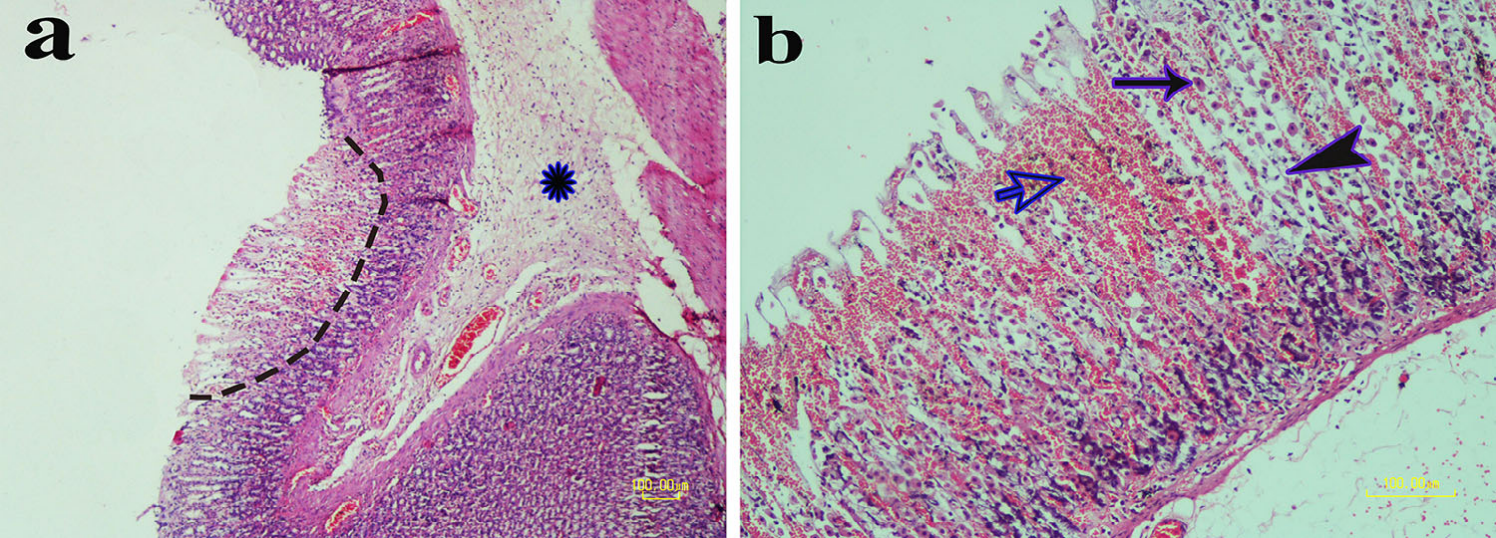
Microscopic features of ethanol‐induced gastric ulcer. (a) Deep necrosis in the gastric mucosa (dotted line) is observed that continues until near the base of gland. Submucosal layer is thickened due to infiltrated inflammatory cells and fibrin deposition (asterisk). (b) Gastric mucosa shows severe intergland haemorrhage (open arrow) accompanied with necrosis and desquamations of epithelial cells (arrowhead). The parietal cells are wrinkled with pyknotic nucleus (arrow) (HE staining, Bar = 100 µm).

In the ethanol group, histopathologic examinations revealed different severities of epithelial sloughing that resulted in erosions and ulcers formation. A fibrinonecrotic layer covered ulcerative lesions superficially. Around the ulcers borders, inflammatory cells especially neutrophils, and a few eosinophils were infiltrated. Necrotic lesions in the deep mucosal layer were associated with leucocyte infiltration. Hyperaemia and haemorrhage occurred in the lamina properia. This group showed significant ulcer scoring based on ulcer depth. Necrotic and ulcerative damages were more extended to the submucosal and fewer to the muscular layers (Figure 5).

**FIGURE 6:**
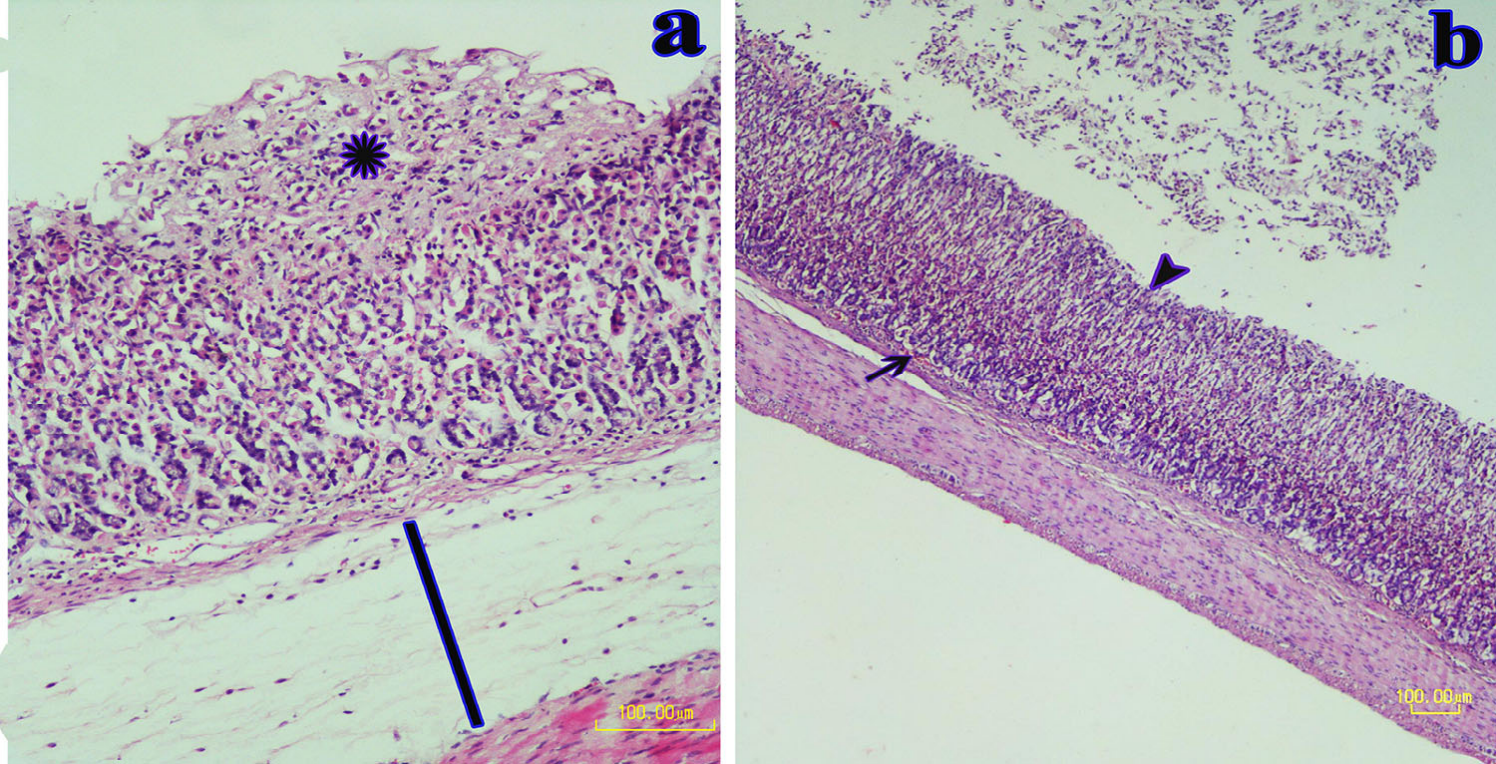
Pretreatment of ethanol‐induced gastric ulcer with ranitidine and donkey's milk. (a) Ranitidine group shows superficial erosion (asterisk) in the one third of the mucosal layer. Tunica submucosa (line) is distended because of edema and mild infiltrated inflammatory cells. (b) In the donkey's milk group, protection of gastric integrity and minimum injuries is obvious. Small erosion is present at the surface of the mucosal layer (arrowhead). Mild hyperaemia (arrow) has occurred in the gastric mucosa. The submucosal layer is not edematous (HE staining, Bar = 100 µm).

Pretreatment with milk and ranitidine preserved the gastric mucosa against ethanol injury. The maximum protective effect was related to donkey milk with mild gastric damage as compared to the ethanol‐treated rats. Few superficial erosions, haemorrhagic foci and mild inflammatory cells were observed in this group (Figure 6). It was also revealed that the histopathologic score of the donkey's milk and ranitidine groups was significantly lower than the ethanol group (Table 2).

**TABLE 2:** A semiquantitative histopathologic scoring system based on gastric ulcer depth.

Groups	Ethanol	Ranitidine	Donkey milk
Histopathologic score	2.2 ± 0.2^a^	0.8 ± 0.37^b^	0.40 ± 0.24^b^

*Note*: Different letters above the data indicate a statistically significant difference (*p* ≤ 0.05).

## 4 Discussion

Functional foods are a large group of foods with potentially positive effects on health. Donkey milk is classified as ‘pharmafood’ due to high its nutritional contents, nourishing and functional properties. In recent years, interest in donkey milk has increased (Garhwal et al. 2022; Tafaro et al. 2007). Donkey milk is rich in essential fats, minerals, protein, bioactive enzymes and various growth factors like riboflavin and vitamin D. Antibacterial properties against a broad range of gram‐positive and gram‐negative bacteria amplify host defence and regulate microbial flora in the gastrointestinal tract. DM supplements prevent asthma (Lu et al. 2021), Type 2 diabetes (Li et al. 2020) and inflammatory bowel diseases such as Crohn's disease (Yvon et al. 2018). Antibacterial compositions such as lysozyme and lactoferrin in donkey milk inhibit the growth of pathogenic bacteria on the skin and reduce the rate of skin infection (Vincenzetti et al. 2011). Formulated milk with some substances is used to treat eczema, psoriasis, acne and some skin diseases. Nowadays, donkey milk is also used in the cosmetics industry such as the production of soap and face cream (Conte and Passantino 2008; Brumini et al. 2016). Donkey milk is rich in vitamins and polyunsaturated fatty acids (Aspri et al., 2016) and contains anti‐ageing, antioxidant and regenerating compounds that hydrate skin and prevent the ageing process.

The protein of donkey milk has almost two equal parts including casein and whey. In cow's milk, casein is five times more than whey (Brumini et al. 2016). Recent studies have revealed that donkeys’ milk may be an undertaking food for infants with cows’ milk protein allergy or multiple food intolerance (Tesse et al. 2009; Garhwal et al. 2022) and also in the elderly due to its capability to up‐regulate the immune response (Tafaro et al. 2007; Amati et al. 2010).

In accordance with the beneficial nutrients in milk, there is an opinion that the consumption of this valuable food is an important element in a healthy and balanced diet. Various bioactive peptides, including digestive health peptides, are present in milk (Martini et al. 2021).

GU is one of the major gastrointestinal disorders. Its incidence increases due to rapid development and civilizational limitations. Therefore, we evaluated the possible protective effect of fresh donkey milk against ethanol‐induced GUs in rat.

In the present study, we investigated the gastroprotective activity of donkey milk on ethanol‐induced GU in rat. We applied ethanol for creation of GU. The ethanol group showed moderate to severe occurrence of erosions, ulcers, hyperaemia, haemorrhage in the mucosal layer, and leucocyte infiltration and fibrinohemorrhagic exudate in the tunica submucosa. Pretreatment with milk and ranitidine decreased the gastric mucosal damages against ethanol injury. The most protective effect was related to donkey milk. In this group, the thickness of ulcerated mucosa was similar to the normal mucosa, and very small superficial erosions and haemorrhagic foci were observed in the gastric wall. Results of our study indicated donkey milk decreased UI significantly compared to the ethanol group. Both the donkey milk and ranitidine reduced markedly gastric mucosal damages produced by ethanol, but the donkey milk group with a higher preventive index was superior to the ranitidine group.

Administration of ethanol in rats produced noticeable GUs in the secreting portion of the stomach. In experimental studies, alcohol is commonly used for induction of GUs in animal models. The pathogenesis of ethanol‐induced GUs is multifactorial. After ingestion, alcohol disturbs the secretory activity of the stomach and leads to depletion of mucus content in the gastric wall. The mucosal barrier is broken as a result of gastric vascular damage. Ethanol activates two major signalling pathways, including inflammation and oxidative stress in the tunica mucosa layer. The severe haemorrhage, necrotizing effects and cell exfoliation of ethanol are related to free radicals production and enhancement of lipid peroxidation (Verma and Kumar 2016; Jabbar et al. 2022).

Oxidative stress is a critical pathogenic agent during gastric ulceration. In the present research, lipid peroxidation in GUs was determined by the measurement of MDA level. Ethanol significantly increased MDA content in gastric tissue, whereas pretreatment with donkey milk and ranitidine successfully decreased MDA production. Our results demonstrated that donkey milk significantly improved oxidative stress in GUs.

GSH is a powerful and the most abundant cellular endogenous antioxidant. GSH is a reduced peptide which can donate an electron and formed oxidized oxidized glutathione (GSSG). Numerous studies revealed that GSH/GSSG redox system is involved in many important cellular functions that include apoptosis, proliferation, cell differentiation, signal transduction, cytokine production and immune responses (Forman 2009; Wu et al. 2004). In the pathogenesis of diseases, a decrease in GSH has been involved (Wu et al. 2004). We investigated GSH activity in stomach tissue in the ethanol‐induced GU of rats and observed reduction in ethanol‐damaged stomach. Donkey's milk was significantly in highest level in compared with the ranitidine and ethanol groups. This result shows preventive effect of it in contrast to the negative effect of ethanol on the gastric tissue. Our experimental results are in line with the previous research studies. Literature review indicates that there is an important relationship between gastric GSH levels and ulcer severity (Dursun et al. 2009).

Few researchers have described the effect of donkey milk on GUs. In consistent with our results, Tastekin et al. (2018) examined the protective effect of donkey milk against indomethacin‐induced GUs. Donkey milk had decreased the total area of erosions and ulcers significantly compared with the indomethacin group. GSH level was increased and MDA decreased significantly in this group. Moreover, expression of tumour necrosis factor‐α was higher and stronger in the indomethacin group, whereas lower expression was observed in the donkey milk group. They showed donkey milk protects gastric mucosa against induced indomethacin injuries via its anti‐inflammatory and antioxidant effects.

Some studies show that whey protein in milk protects the stomach wall (Marshall 2004; Rosaneli et al. 2004). Yoo et al. (2018) investigated the gastroprotective effect of fresh bovine milk on GUs induced by acidified ethanol (HCl‐ethanol) in a mouse model. Mice received different doses of commercial fresh bovine milk orally for 14 days, and 1 h after the last oral administration of bovine milk, GU was induced. Their results showed that pretreatment with bovine milk significantly suppressed the development of lesions in gastric mucosa and increased catalase, superoxide dismutase (SOD) and mucus contents in the stomach. Administration of bovine milk had increased nitrate/nitrite levels. In addition, the MDA level and expression of proinflammatory genes, including transcription factor NF‐κB, cyclooxygenase‐2 and inducible NO synthase in the stomach, decreased in animals. They suggested that bovine milk can prevent the formation of gastric damage caused by acid‐alcohol in mice. Matsumoto et al. (2001) reported that α‐LA, one of the major proteins in milk, inhibits gastric damages due to the synthesis of prostaglandin, elevation of gastric luminal pH, increasing the mucin in both gastric fluid and adhered mucus gel to the gastric wall.

The exact protective and/or therapeutic mechanism of donkey milk is unknown. This milk possesses natural protective antimicrobial factors such as high lysozyme content and a specific epidermal growth factor (EGF). These compounds are believed to have a positive impact on the health and integrity of the gastrointestinal mucosa. This is especially valuable for individuals with weakened immune systems, such as children, the elderly and patients recovering from illness (Scafizzari et al. 2009). Antimicrobial properties of donkey milk suppress bacteria and viruses related to intestinal infections (Šarić et al. 2012; Brumini et al. 2013). Antimicrobial action of milk lactoperoxidase exerts its role through the oxidation of thiocyanate ions by hydrogen peroxide that both of them are present in biological fluids and also in milk (Vincenzetti et al. 2012).

Recently, positive effects of donkey milk in the regulation of immune response have been shown (Jirillo, Jirillo, and Magrone 2010). This valuable substance induces the production of NO and cytokines particularly IL‐8, IL‐6, IL‐1b and TNF‐α for stimulating immunity. NO causes dilation of blood vessels that results in blood flow improvement and decreased blood pressure (Amati et al. 2010; Yvon et al. 2018).

In current research, ranitidine enhanced the gastroprotective activity against ethanol‐induced lesions in rat. In addition, we observed a noticeable reduction of MAD in gastric tissue by ranitidine rather than ethanol‐ulcerated stomach. In this study, the gastroprotective effect of ranitidine may be related to its antioxidant properties and ability to decrease lipid peroxidation. Ahmadi et al. (2011) reported that the therapeutic effect of ranitidine on GUs could be related to its antioxidant capacity and scavenging of hydroxyl radical. In the study of (Sharif and Dugani 2013), ranitidine (50 mg/kg) significantly amplified the gastric mucin content as compared to the ethanol‐treated group. The secreted gastric mucins from specialized mucous cells play an important role in the protection of gastric mucosa from acid‐peptic injury.

In the present study, ethanol administration in rats destructed gastric mucosa. However, pretreatment with donkey milk and ranitidine had protective effects against ethanol‐gastric damage and increased significantly the inhibition index of ulcers in the treated rats. The donkey milk displayed better protection against ulceration compared to ranitidine‐treated rats. Our results demonstrated that MDA levels in gastric tissue decreased significantly in both pretreated groups rather than the ethanol‐treated rats. MDA is a marker for lipid peroxidation.

## 5 Conclusion

In conclusion, donkey milk has a protective role and keeps gastric mucosa from induced injury by ethanol. This valuable food reduced UI more than ranitidine as a standard drug. Donkey milk may exert its protective property by activation of antioxidant mechanisms such as GSH and MDA. However, the precise mechanism for this action remains to be elucidated. Antioxidant compounds may play an important role in GU therapy by scavenging free radicals. Natural products are considered as alternative therapies instead of chemical drugs with side effects. Several natural products have been shown to have anti‐ulcerogenic effects due to their antioxidant properties. Scavenging of reactive oxygen species is a new strategy to prevent and treat chronic and degenerative diseases such as peptic ulcer (Fahmy, Amer, and Al‐killidar 2015; Sayed et al. 2016). Thereby, the present study suggests that one of the anti‐ulcerogenic efficacies of donkey milk may be related to its antioxidative properties.

## Author Contributions

Masoud Sami contributed to the conception and design of the study. Shahrzad Azizi designed the study, pathologic investigation, writing–review and editing the manuscript. Reza Kheirandish designed the study and evaluation of the pathologic results. Hadi Ebrahimnejad and Shiva Alizadeh contributed to the laboratory techniques.

## Ethics Statement

All animal investigations were carried out on the basis of the Guide for Care and Use of Laboratory Animals published by the National Institutes of Health (NIH publication No. 85‐23, revised 1985). The study was approved by the Institutional Animal Care and Use Committee of our veterinary school.

## Conflicts of Interest

The authors declare no conflicts of interest.

### Peer Review

The peer review history for this article is available at https://publons.com/publon/10.1002/vms3.70156.

## Data Availability

The datasets of this study are not shared. Research data are available from the corresponding author upon reasonable request.

## References

[vms370156-bib-0001] Ahmadi, A. , M. A. Ebrahimzadeh , S. Ahmad‐Ashrafi , M. Karami , M. R. Mahdavi , and S. S. S. Saravi . 2011. “Hepatoprotective, Antinociceptive and Antioxidant Activities of Cimetidine, Ranitidine and Famotidine as Histamine H2 Receptor Antagonists.” Fundamental and Clinical Pharmacology 25, no. 1: 72–79. 20070855 10.1111/j.1472-8206.2009.00810.x

[vms370156-bib-0002] Al Asmari, A. , H. Al Shahrani , N. Al Masri , A. Al Faraidi , I. Elfaki , and M. Arshaduddin . 2016. “Vanillin Abrogates Ethanol Induced Gastric Injury in Rats via Modulation of Gastric Secretion, Oxidative Stress and Inflammation.” Toxicology Reports 3: 105–113. 28959528 10.1016/j.toxrep.2015.11.001 PMC5615375

[vms370156-bib-0003] Amati, L. , G. Marzulli , M. Martulli , et al. 2010. “Donkey and Goat Milk Intake and Modulation of the human Aged Immune Response.” Current Pharmaceutical Design 16: 864–869. 20388099 10.2174/138161210790883651

[vms370156-bib-0004] Andrews, F. M. , C. R. Reinemeyer , M. D. Mccracken , et al. 2002. “Comparison of Endoscopic, Necropsy and Histology Scoring of Equine Gastric Ulcers.” Equine Veterinary Journal 34, no. 5: 475–478. 12358050 10.2746/042516402776117827

[vms370156-bib-1004] Aspri, M. , N. Economou , P. Papademas . 2016. “Donkey Milk: An Overview on Functionality, Technology, and Future Prospects.” Food Reviews International 33, no. 3: 316–333.

[vms370156-bib-0005] Brumini, D. , A. Criscione , S. Bordonaro , G. E. Vegarud , and D. Marletta . 2016. “Whey Proteins and Their Antimicrobial Properties in Donkey Milk: A Brief Review.” Dairy Science and Technology 96, no. 1: 1–14.

[vms370156-bib-0006] Brumini, D. , C. B. Furlund , I. Comi , et al. 2013. “Antiviral Activity of Donkey Milk Protein Fractions on Echovirus Type 5.” International Dairy Journal 28, no. 2: 109–111.

[vms370156-bib-0007] Chen, H. , H. Liao , Y. Liu , et al. 2015. “Protective Effects of Pogostone From *Pogostemonis herba* Against Ethanol‐induced Gastric Ulcer in Rats.” Fitoterapia 100: 110–117. 25481373 10.1016/j.fitote.2014.11.017

[vms370156-bib-0008] Conte, F. , and A. Passantino . 2008. “Isolation of *Enterobacter sakazakii* From Ass' Milk in Sicily: Case Report, Safety and Legal Issues.” Travel Medicine and Infectious Disease 6, no. 4: 250–252. 18571118 10.1016/j.tmaid.2008.01.003

[vms370156-bib-0009] de Lira Mota, K. S. , G. E. N. Dias , M. E. F. Pinto , et al. 2009. “Flavonoids With Gastroprotective Activity.” Molecules (Basel, Switzerland) 14, no. 3: 979–1012. 19305355 10.3390/molecules14030979 PMC6253827

[vms370156-bib-0010] Dursun, H. , M. Bilici , F. Albayrak , et al. 2009. “Antiulcer Activity of Fluvoxamine in Rats and Its Effect on Oxidant and Antioxidant Parameters in Stomach Tissue.” BMC Gastroenterology 9: 36. 19457229 10.1186/1471-230X-9-36 PMC2693117

[vms370156-bib-0011] Fahmy, S. R. , M. A. Amer , and M. H. Al‐killidar . 2015. “Ameliorative Effect of the Sea Cucumber *Holothuria arenicola* Extract Against Gastric Ulcer in Rats.” Journal of Basic and Applied Zoology 72: 16–25.

[vms370156-bib-0012] Forman, H. J. 2009. “Glutathione in Health and Disease.” Molecular Aspects of Medicine 30: 1–110. 18796312 10.1016/j.mam.2008.08.006 PMC2696075

[vms370156-bib-0013] Garhwal, R. , K. Sangwan , R. Mehra , et al. 2022. “A Systematic Review of the Bioactive Components, Nutritional Qualities and Potential Therapeutic Applications of Donkey Milk.” Journal of Equine Veterinary Science 115: 104006. 35526725 10.1016/j.jevs.2022.104006

[vms370156-bib-0014] Guo, H. Y. , K. Pang , X. Y. Zhang , et al. 2007. “Composition, Physiochemical Properties, Nitrogen Fraction Distribution, and Amino Acid Profile of Donkey Milk.” Journal of Dairy Science 90, no. 4: 1635–1643. 17369203 10.3168/jds.2006-600

[vms370156-bib-0015] Hajrezaie, M. , S. Golbabapour , P. Hassandarvish , et al. 2012. “Acute Toxicity and Gastroprotection Studies of a New Schiff Base Derived Copper (II) Complex Against Ethanol‐induced Acute Gastric Lesions in Rats.” PLoS ONE 7, no. 12: e51537. 23251568 10.1371/journal.pone.0051537 PMC3519725

[vms370156-bib-0016] Jabbar, A. A. , F. O. Abdullah , K. Abdoulrahman , et al. 2022. “Gastroprotective, Biochemical, and Acute Toxicity Effects of *Papaver decaisnei* Against Ethanol‐induced Gastric Ulcers in Rats.” Processes 10, no. 10: 1985.

[vms370156-bib-0017] Jirillo, F. , E. Jirillo , and T. Magrone . 2010. “Donkey's and Goat's Milk Consumption and Benefits to Human Health With Special Reference to the Inflammatory Status.” Current Pharmaceutical Design 16, no. 7: 859–863. 20388098 10.2174/138161210790883688

[vms370156-bib-1018] Laine, L. , K. Takeuchi , and A. Tarnawski . 2008. “Gastric Mucosal Defense and Cytoprotection: Bench to Bedside.” Gastroenterology 135, no. 1: 41–60. 18549814 10.1053/j.gastro.2008.05.030

[vms370156-bib-0018] Li, Y. , Y. Fan , A. S. Shaikh , Z. Wang , D. Wang , and H. Tan . 2020. “Dezhou Donkey (*Equus asinus*) Milk a Potential Treatment Strategy for Type 2 Diabetes.” Journal of Ethnopharmacology 246: 112221. 31494203 10.1016/j.jep.2019.112221

[vms370156-bib-0019] Lu, Y. , Y. Zhou , Y. Lin , et al. 2021. “Preventive Effects of Donkey Milk Powder on the Ovalbumin‐induced Asthmatic Mice.” Journal of Functional Foods 84: 104603.

[vms370156-bib-0020] Mansueto, P. , G. Iacono , G. Taormina , et al. 2013. “Ass's Milk in Allergy to Cow's Milk Protein: A Review.” Acta Medica Mediterranea 29: 153–160.

[vms370156-bib-0021] Marshall, K. 2004. “Therapeutic Applications of Whey Protein.” Alternative Medicine Review 9, no. 2: 136–156. 15253675

[vms370156-bib-0022] Martini, M. , I. Altomonte , D. Tricò , R. Lapenta , and F. Salari . 2021. “Current Knowledge on Functionality and Potential Therapeutic Uses of Donkey Milk.” Animals (Basel) 11, no. 5: 1382. 34067986 10.3390/ani11051382 PMC8152225

[vms370156-bib-0023] Matsumoto, H. , Y. Shimokawa , Y. Ushida , T. Toida , and H. Hayasawa . 2001. “New Biological Function of Bovine α‐Lactalbumin: Protective Effect Against Ethanol‐and Stress‐induced Gastric Mucosal Injury in Rats.” Bioscience, Biotechnology, and Biochemistry 65, no. 5: 1104–1111. 11440124 10.1271/bbb.65.1104

[vms370156-bib-0024] Okayama, T. , N. Yoshida , K. Uchiyama , T. Takagi , H. Ichikawa , and T. Yoshikawa . 2009. “Mast Cells Are Involved in the Pathogenesis of Indomethacin‐induced Rat Enteritis.” Journal of Gastroenterology 44, no. 19: 35–39. 19148791 10.1007/s00535-008-2267-5

[vms370156-bib-0025] Ononye, S. N. , M. D. VanHeyst , E. Z. Oblak , et al. 2013. “Tropolones as Lead‐Like Natural Products: The Development of Potent and Selective Histone Deacetylase Inhibitors.” ACS Medicinal Chemistry Letters 4, no. 8: 757–761. 24900743 10.1021/ml400158k PMC4027479

[vms370156-bib-0026] Ozbayer, C. , H. Kurt , Z. Ozdemir , et al. 2014. “Gastroprotective, Cytoprotective and Antioxidant Effects of *Oleum cinnamomi* on Ethanol Induced Damage.” Cytotechnology 66, no. 3: 431–441. 23868387 10.1007/s10616-013-9594-y PMC3973792

[vms370156-bib-0027] Ramakrishnan, K. , and R. C. Salinas . 2007. “Peptic Ulcer Disease.” American Family Physicians 76, no. 7: 1005–1012. 17956071

[vms370156-bib-0028] Ritter, J. M. , E. Robinson , J. Fullerton , et al. 2014. “Rang & Dale's Pharmacology.” Elsevier Health Sciences 244–259.

[vms370156-bib-0029] Riddles, P. W. , R. K. Andrews , R. L. Blakeley , and B. Zerner . 1983. “ *Jack bean* urease VI. Determination of Thiol and Disulfide Content: Reversible Inactivation of the Enzyme by the Blocking of the Unique Cysteine Residue.” Biochimica Et Biophysica Acta (BBA)—Protein Structure and Molecular Enzymology 743, no. 1: 115–120.

[vms370156-bib-0030] Rosaneli, C. F. , A. E. Bighetti , M. A. Antônio , J. E. Carvalho , and V. C. Sgarbieri . 2004. “Protective Effect of Bovine Milk Whey Protein Concentrates on the Ulcerative Lesions Caused by Subcutaneous Administration of Indomethacin.” Journal of Medicinal Food 7, no. 3: 309–314. 15383224 10.1089/jmf.2004.7.309

[vms370156-bib-0031] Sabiu, S. , T. Garuba , T. Sunmonu , et al. 2015. “Indomethacin‐Induced Gastric Ulceration in Rats: Protective Roles of *Spondias mombin* and *Ficus exasperata* .” Toxicology Reports 2: 261–267. 10.1016/j.toxrep.2015.01.002. 28962358 PMC5598261

[vms370156-bib-0032] Šarić, L. Ć. , B. M. Šarić , A. I. Mandić , et al. 2012. “Antibacterial Properties of Domestic Balkan Donkeys' Milk.” International Dairy Journal 25, no. 2: 142–146.

[vms370156-bib-0033] Saxena, B. , and S. Singh . 2011. “Investigations on Gastroprotective Effect of citalopram, an Antidepressant Drug Against Stress and Pyloric Ligation Induced Ulcers.” Pharmacological Reports 63, no. 6: 1413–1426. 22358089 10.1016/s1734-1140(11)70705-8

[vms370156-bib-0034] Sayed, D. A. , S. R. Fahmy , A. M. Soliman , and N. S. Hussein . 2016. “Antiulcerogenic Efficacy of Ethanolic Extract of *Vitis vinifera* Leaves in Rats.” International Journal of Pharmacy and Pharmaceutical Sciences 8, no. 9: 163–172.

[vms370156-bib-0035] Scafizzari, M. , F. Giannico , O. Potere , et al. 2009. “Epidermal Growth Factor (EGF) in Mare and Ass Milk: A Preliminary Investigation.” Italian Journal of Animal Sciences 8, no. S2: 737.

[vms370156-bib-0036] Sharif, Z. , and A. Dugani . 2010. “Potentiation of the Gastroprotective Effect of Ranitidine by Verapamil in Ethanol‐induced Ulcer in Rats.” International Journal of Pharmaceutical and Biological Archives 4, no. 4: 696–705.

[vms370156-bib-0037] Tafaro, A. , T. Magrone , F. Jirillo , et al. 2007. “Immunological Properties of Donkey's Milk: Its Potential Use in the Prevention of Atherosclerosis.” Current Pharmaceutical Design 13, no. 36: 3711–3717. 18220810 10.2174/138161207783018590

[vms370156-bib-0038] Tastekin, E. , S. Ayvaz , U. Usta , N. Aydogdu , E. Cancilar , and F. O. Puyan . 2018. “Indomethacin‐Induced Gastric Damage in Rats and the Protective Effect of Donkey Milk.” Archives of Medical Science: AMS 14, no. 3: 671–678. 29765456 10.5114/aoms.2016.59645 PMC5949905

[vms370156-bib-0039] Tesse, R. , C. Paglialunga , S. Braccio , and L. Armenio . 2009. “Adequacy and Tolerance to Ass's Milk in an Italian Cohort of Children With Cow's Milk Allergy.” Italian Journal of Pediatrics 35, no. 1: 1–4. 19589131 10.1186/1824-7288-35-19 PMC2717565

[vms370156-bib-0040] Turner, P. V. , T. Brabb , C. Pekow , and M. A. Vasbinder . 2011. “Administration of Substances to Laboratory Animals: Routes of Administration and Factors to Consider.” Journal of American Association for Laboratory Animal Science 50, no. 5: 600–613. PMC3189662 22330705

[vms370156-bib-0041] Valko, M. , D. Leibfritz , J. Moncol , M. T. Cronin , M. Mazur , and J. Telser . 2007. “Free Radicals and Antioxidants in Normal Physiological Functions and Human Disease.” International Journal of Biochemistry and Cell Biology 39, no. 1: 44–84. 16978905 10.1016/j.biocel.2006.07.001

[vms370156-bib-0042] Verma, S. , and V. L. Kumar . 2016. “Attenuation of Gastric Mucosal Damage by Artesunate in Rat: Modulation of Oxidative Stress and NFκB Mediated Signaling.” Chemico‐Biological Interactions 257: 46–53. 27474069 10.1016/j.cbi.2016.07.027

[vms370156-bib-0043] Vincenzetti, S. , A. Amici , S. Pucciarelli , et al. 2012. “A Proteomic Study on Donkey Milk.” Biochemical and Analytical Biochemistry 1, no. 109: 2161–2209.

[vms370156-bib-0044] Vincenzetti, S. , M. Savini , C. Cecchini , et al. 2011. “Effects of Lyophilization and Use of Probiotics on Donkey's Milk Nutritional Characteristics.” International Journal of Food Engineering 7, no. 5: 1–14.

[vms370156-bib-0045] Wu, G. , Y. Z. Fang , S. Yang , J. R. Lupton , and N. D. Turner . 2004. “Glutathione Metabolism and Its Implications for Health.” Journal of Nutrition 134: 489–492. 14988435 10.1093/jn/134.3.489

[vms370156-bib-0046] Yoo, J. H. , J. S. Lee , Y. S. Lee , S. Ku , and H. J. Lee . 2018. “Protective Effect of Bovine Milk Against HCl and Ethanol–Induced Gastric Ulcer in Mice.” Journal of Dairy Science 101, no. 5: 3758–3770. 29477532 10.3168/jds.2017-13872

[vms370156-bib-0047] Yvon, S. , M. Olier , M. Leveque , et al. 2018. “Donkey Milk Consumption Exerts Anti‐inflammatory Properties by Normalizing Antimicrobial Peptides Levels in Paneth's Cells in a Model of Ileitis in Mice.” European Journal of Nutrition 57, no. 1: 155–166. 27581119 10.1007/s00394-016-1304-z

